# DSM-5 Changes in Attention Deficit Hyperactivity Disorder and Autism Spectrum Disorder: Implications for Comorbid Sleep Issues

**DOI:** 10.3390/children4080062

**Published:** 2017-07-27

**Authors:** Ujjwal P. Ramtekkar

**Affiliations:** 1 Department of Psychiatry, Compass Health Network, Wentzville, MO 63385, USA; uramtekkar@compasshn.org; 2 Division of Child and Adolescent Psychiatry, University of Missouri-Columbia School of Medicine, 1 Hospital Drive, Columbia, MO 65212, USA

**Keywords:** sleep, ADHD, autism, DSM-5, comorbidity, neurodevelopmental disorders

## Abstract

Attention Deficit Hyperactivity Disorder (ADHD) and Autism Spectrum Disorder (ASD) are the most common neurodevelopmental disorders. Despite significant comorbidity, the previous diagnostic criteria prohibited the simultaneous diagnosis of both disorders. Sleep problems are highly prevalent in both disorders; however, these have been studied independently for ADHD and ASD. In the context of revised criteria in the Diagnostic Statistical Manual of Mental Disorders 5th edition (DSM-5) that allows combined diagnosis of ADHD and ASD, this short review presents an overview of relationship between sleep problems, ADHD and ASD, as well as conceptualizing the shared pathophysiology. The practical considerations for clinical management of sleep problems in combination with ADHD and ASD are also discussed.

## 1. Introduction

Neurodevelopmental disorders consist of a group of disorders with onset in the early development period and are characterized by developmental deficits in several functional domains. Attention Deficit Hyperactivity Disorder (ADHD) and Autism Spectrum Disorder (ASD) are among the most prevalent neurodevelopmental disorders. ADHD is characterized by deficits in attention, organization, activity levels and impulse control and is classified into three subtypes—inattentive presentation, hyperactive-impulsive presentation and combined presentation [1]. Large epidemiological studies have demonstrated that the prevalence is higher in the community-based, non-referred population, and it continues to cause functional impairment with increasing age, even if the total number of symptoms decreases over time [2]. Autism spectrum disorder is characterized by persistent deficits in social communication and social interaction across multiple contexts (deficits in social-emotional reciprocity, non-verbal communication and developing relationships), as well as restricted, repetitive patterns of behavior, interests, or activities [1]. The prevalence of ASD has been on the rise in the past decade and the most recent estimate by Center for Disease Control indicates 1 in 68 children were identified with ASD [3].

Prior to the recent revision of Diagnostic Statistical Manual of Mental Disorders (DSM), 5th edition, published in 2013, the diagnostic criteria for ADHD and ASD did not allow the simultaneous diagnosis in individuals, despite significant comorbidity. Studies indicate that 15–25% of youth with ADHD meet the criteria for ASD, whereas 50–70% of those with ASD have comorbid ADHD [4]. There is well-established research highlighting the high prevalence of a wide variety of sleep problems in ADHD and ASD [4,5,6]. However, the research studies have traditionally followed the previous diagnostic criteria and have often excluded the possibility of co-occurrence of ASD and ADHD while studying the associated sleep problems and developing treatment strategies. In the context of the changes in diagnostic criteria, this short review attempts to conceptualize and discuss shared pathophysiology, and practical considerations in management of sleep issues in comorbid ADHD and ASD.

## 2. Sleep Issues in ADHD and ASD

Both ADHD and ASD have a high prevalence of comorbid sleep issues across major categories in the International Classification of Sleep Disorders, 3rd edition (ICSD-3); mainly insomnia, circadian rhythm sleep-wake disorders, sleep-related movement disorders, leep-related breathing disorders (SRBD) and, to a lesser extent, parasomnias. The details are discussed later in the section on shared pathophysiology. Children and adolescents with ADHD are found to have a variety of sleep problems with a prevalence in the range of 50–74%, whereas the percentage of comorbid sleep issues is even higher—up to 80%—in children with ASD [5,6,7]. There have been several studies using subjective measures (questionnaires and parent reports) and objective investigations (actigraphy and polysomnography) to explore the association of sleep problems as comorbid sleep disorders, as well as the expression of intrinsic pathology unique to the disorder. 

### 2.1. Subjective Studies in Sleep and ADHD

There have been several studies using subjective parent report rating scales, questionnaires and screening instruments to investigate sleep problems in ADHD. The results have been fairly consistent for the high prevalence of sleep onset insomnia [8]. Large population-based studies using parent report or combined parent and teacher report indicated that the rates of sleep issues were higher in ADHD when compared to those without ADHD, and that ADHD was associated most commonly with sleep onset insomnia, bedtime anxiety, intermittent awakenings, sleep-related disordered breathing, parasomnias and daytime sleepiness or napping [9,10]. In a meta-analysis of subjective studies of sleep in ADHD, there were six main parent-reported sleep issues that were significantly higher. The most common impairment was bedtime resistance, followed by difficulty with morning awakenings, sleep onset difficulties, sleep-disordered breathing, night awakenings and daytime sleepiness [7]. 

### 2.2. Objective Studies in Sleep and ADHD

Objective studies using polysomnography (PSG) have been inconsistent across various sleep parameters. In agreement with the subjective studies, objective studies have identified longer sleep onset latency and increased Rapid Eye Movement (REM) sleep latency on PSG [5,11,12]. The most consistent associations were found with high apnea/hypopnea index and nocturnal motor activity, i.e., periodic limb movements in sleep (PLMS) in children with ADHD [13]. Several studies have reported changes in the REM sleep percentage in ADHD. However, some studies report increased REM sleep while other studies report decreased REM sleep. The inconsistencies are often attributed to the changes in REM across developmental stages due to maturation as well as night-to-night variability. There appears to be significant discrepancy regarding total sleep time between usage of PSG and actigraphy. While some studies report an increase in total sleep time in ADHD, others report a decrease in total sleep time due to intermittent awakenings or no difference between ADHD and controls without ADHD [12,14,15].

### 2.3. Subjective Studies in Sleep and Autism

Like ADHD, sleep onset insomnia is among the most common sleep problems in ASD. Several studies have also reported sleep maintenance insomnia, shortened sleep time, bedtime resistance and sleep related anxiety as common sleep issues in ASD [6,16,17]. While there have been reports of parasomnias such as sleepwalking, nightmares, night terrors, and night time crying, these appear to be less common in children with ASD. On the other hand, night time disruptive behaviors such as grunting, laughing, and head banging were more commonly present than in controls on night time arousal in children with ASD [18,19,20,21].

### 2.4. Objective Studies in Sleep and Autism

Unlike ADHD, there is agreement between the subjective and objective measures of sleep in ASD. The objective studies using PSG reported an increase in sleep latency, a decrease in sleep efficiency, a decrease in total sleep time and a decrease in REM latency [22,23]. Other studies have also reported a greater percentage of slow-wave sleep and a lower percentage of REM sleep [24,25]. Interestingly, there are also reports of REM behavior disorders in children with night time arousals. However, those were mostly attributed to medication treatment. Sleep onset insomnia continued to be the most prevalent sleep problem in children with ASD on actigraphy studies [26]. 

### 2.5. Differential Impact of Combined ADHD and ASD on Sleep

Presence of comorbid ADHD and ASD symptoms have been shown to have a greater impact on behavioral, emotional and cognitive difficulties than with the presence of ADHD or ASD alone [27]. There is a scarcity of studies using objective sleep measures in children with comorbid ASD and ADHD. However, studies using parental reports have indicated that sleep problems are more pronounced in comorbid ADHD and ASD [28,29]. Regarding the phenotype, sleep problems in comorbid ADHD and ASD more closely resemble those with ADHD than ASD. It has been postulated that unique sleep difficulties in combined ADHD and ASD are attributed to hyperactivity [6]. Along the same lines, externalizing behaviors similar to ADHD are also associated with decreased sleep efficiency in children with ASD [30]. Children with combined ADHD and ASD have more sensory and motor deficits, more robust developmental delay, severe impairment in adaptive functions, and lower intelligence quotient (IQ), which in combination result in ‘additive’ effects, leading to an increase in overall functional impairment. [4]. These deficits in turn impact the prevalence and severity of sleep problems in children with ADHD and ASD.

## 3. Shared Pathophysiology of Sleep Problems in ADHD and ASD

Sleep problems in ADHD and ASD appear to be bidirectional in nature and multifactorial with regard to causes. It is also speculated that the presence of sleep problems is an expression of the intrinsic deficits of these disorders. However, given the similarities in the nature of sleep problems in each disorder and their increased severity in combined ADHD and ASD, common underlying pathophysiology may be at play.

### 3.1. Insomnia and Circadian Rhythm Disorders

Melatonin is a neurohormone, and its metabolites are responsible for circadian physiology, particularly in sleep initiation and maintenance. It is assumed that alterations in melatonin are responsible for insomnia-related disorders. Abnormal circadian pattern of melatonin secretion, particularly dim light melatonin onset (DLMO), have been reported in both ASD and ADHD [31,32]. In addition, CLOCK genes, which are associated with maintenance of circadian rhythm, have been linked to ASD and ADHD as causative factors for core sleep disturbances [33,34]. In addition to the melatonergic system, increased orexinergic system activity and reduced serotonin (5-HT) activity have been presumed to be involved in insomnia in both ADHD and ASD [35]. 

Environmental factors, such as exposure to light, meal times, social cues and bedtime rituals, also play a significant role in the initiation of sleep [36]. It is observed that children with ASD and ADHD have faulty transmission of entrainment cues, disorganized behaviors, and an inability to transition from state of stimulus-seeking alertness to the passive state of sleep leading to sleep onset insomnia [6,37]. 

Medications, such as stimulants used to treat symptoms of hyperactivity/impulsivity, or other psychotropics used to treat comorbid psychiatric symptoms such as anxiety, may also interfere and prolong sleep onset latency [38,39].

### 3.2. Sleep-Related Movement Disorders

Restless Leg Syndrome (RLS) and Periodic Limb Movement Disorder (PLMD) are more common in both ASD and ADHD than the common population [19,40,41]. Due to the close association with low serum ferritin, it is assumed that sensory aversion, poor eating habits, and disorganized behaviors in children with ADHD and ASD promote the development of nutritional deficiencies. Appetite suppression leading to poor food intake is also a side effect of stimulant medication used to treat ADHD symptoms, and may contribute to RLS and/or PLMD. The sleep-related movement disorders are often associated with poor sleep efficiency, sleep onset and maintenance insomnia and daytime sleepiness. Unfortunately, it is challenging to clinically diagnose RLS and PLMD in children with ASD and ADHD due to reliance on the self-report of symptoms, combined with the fact that these youths often have communication deficits.

### 3.3. Sleep-Related Breathing Disorders

Obstructive Sleep Apnea (OSA) and Sleep-Disordered Breathing (SDB) are more prevalent in ADHD and ASD than in the normal population [42]. Studies have indicated that the presence of SRBD has a significant impact on the worsening of the core symptoms of ADHD and ASD, and the correction of SRBD leads to an improvement in daytime functioning [43,44]. The risk factors for SRBD include motor delays, low muscle tone, and obesity, which are common in both ADHD and ASD, but more prevalent in combined ADHD and ASD [4]. Obesity could also be secondary to abnormal or restrictive eating habits, and the use of common medications such as atypical antipsychotics for the treatment of irritability and aggression in children with ASD and ADHD.

### 3.4. Parasomnias

The etiology of parasomnias in ADHD and ASD is complex, but is largely related to increased fragmentation of sleep, changes in non-Rapid Eye Movement NREM sleep and rarity of REM sleep. The behaviors suggestive of parasomnias often include night-terrors, confusional arousals, wake screaming, increased motor activity (sleepwalking) and enuresis, and these have been reported in both disorders [6,12,45]. Although nightmares are not as frequently reported, it is possible that the communication difficulties of these children could interfere with the ability to express their experiences. There have been rare cases of REM behavior disorders reported in both disorders, but these have largely been attributed to medications. Studies have reported that the cyclic alternating pattern, which is an endogenous rhythm recurring in NREM sleep and often used as a measure of NREM stability, is significantly lower in ADHD and ASD [23,46]. In addition, the associated medication use and psychiatric comorbidities such as anxiety also increase the risk of parasomnias.

## 4. Considerations in Treatment of Sleep Problems

Despite the common sleep problems and their shared pathophysiology, there are unique aspects of ADHD as well as ASD that are intrinsic to the disorder. The treatment approaches to sleep problems in combined ADHD and ASD should consider these common and unique factors before implementing the interventions proven effective for either of the disorder. Using traditional interventions for typical developing children without considering the underlying core deficits in ADHD and ASD may not be effective, and could possibly worsen the sleep issues.

### 4.1. Non-Pharmacological Interventions

#### 4.1.1. Sleep Hygiene

Standard sleep hygiene practices for children have been effective in ADHD as well ASD. These include a regular sleep-wake schedule, calming bedtime routines, structured transition to sleep, avoidance of caffeine, large amounts of liquids, naps, exercise immediately before bedtime, avoidance of electronic media soon before bedtime, using the bed only for sleeping and consistent place/bed for sleeping [47,48]. In children with hyperactivity, it is important to emphasize the need to avoid behavioral stimulation that may lead to motor activation and difficulty in ‘settling down’, subsequently leading to sleep fragmentation. Calming routines are of prime importance to assist children with effective transition from wakefulness to sleep. In children with non-functional ritualistic behaviors and stereotypies due to autism, it may be important to ensure environmental factors such as room temperature, furniture alignment and transitional objects for soothing rituals.

#### 4.1.2. Sensory and Behavioral Interventions

Children with combined ADHD and ASD appear to have more severe sensory and motor deficits [4]. Certain sleep problems such as sleep onset insomnia and sleep maintenance insomnia may be related to difficulties with sensory modulation, particularly when children tend to be easily over-aroused by sensory stimuli [49]. Based on the nature of sensory modulation difficulties, it would be beneficial to utilize sensory based interventions for sleep. Use of weighted blankets or specific tools such as ball-blankets, which stimulate sensory receptors and transmit inhibitory signals to the central nervous system, have been shown to reduce the sleep onset latency, intermittent awakenings and intra-individual variability in sleep parameters [50]. In addition to sensory interventions, depending on the individual circumstance and cognitive ability of the child, the established behavioral interventions, such as graduated extinction and bedtime fading, can be implemented for addressing bedtime resistance and sleep onset insomnia [51].

#### 4.1.3. Parent Education and Support

Parents of children with sleep problems have high levels of stress and poor sleep. In addition, younger parent age and presence of sleep problems in caregivers also predict sleep problems in children with combined ADHD and ASD [52]. The bidirectional effect of parental stress and sleep problems in children has been observed in ADHD and ASD [26,53,54]. Addressing the parental stress and providing parental education and training is therefore important for both risk reduction and effective implementation of behavior interventions for sleep problems in children. 

### 4.2. Pharmacological Interventions

There are no U.S. Food and Drug Administration (FDA) approved medications for treatment of sleep problems. However, there have been some studies demonstrating efficacy of some pharmacological agents in treating insomnia in both ASD and ADHD populations. Medication management is often used in combination with behavioral and non-pharmacological interventions. Evidence-based interventions and studies for ADHD and ASD are discussed below. 

#### 4.2.1. Melatonin

Synthetic melatonin has been used for insomnia in ASD and ADHD. Studies have reported that melatonin has been efficacious as a hypnotic, as well as as a chronobiotic, and is well tolerated in both populations. Several randomized controlled trials of melatonin in ASD demonstrated that treatment with melatonin was associated with increased sleep duration and decreased sleep latency [55,56]. The studies of melatonin treatment in children with ADHD also demonstrated similar results [57,58]. The effect of melatonin was noted to be sustained on long-term follow up on both populations. Treatment with melatonin in these studies was also associated with side effects, most commonly bedwetting, headaches, nightmares, daytime sedation and abdominal pain or constipation. The side effect profile should be considered in the context of comorbid disorders that may worsen with the use of melatonin. Although long term effects of melatonin treatment, including potential hypothalamic-pituitary-adrenal (HPA) axis suppression and subsequent growth suppression, has been hypothesized, these concerns are not substantiated, and chronic melatonin use has been deemed safe in children with ADHD [58]. When used as a hypnotic for insomnia, melatonin should be used at lower doses for younger children to avoid side effects. While there are very few dosing studies, clinically the dosing schedule may include 1 mg for younger children, 2–3 mg for school age children and 6–9 mg for adolescents [57,59]. It should be noted that there is no data on increased efficacy with higher doses, but it does pose the risk of increased side effects. When used as a chronobiotic in delayed sleep phase, low dose melatonin between 1–2 mg is administered about 4–5 h before intended bedtime. Melatonin is also available in liquid form, and can be considered for children with sensory hypersensitivities. Studies have often used short-acting as well as longer-acting preparations of melatonin in children with ADHD and ASD. Clinically, short-acting preparations are used for sleep initiation, and long acting-preparations are used for sleep maintenance [60].

#### 4.2.2. Clonidine

Clonidine is an alpha agonist. Limited open label and retrospective studies of clonidine in ADHD and ASD have reported reductions in sleep latency and night time awakenings at doses of between 0.05–0.08 mg [61,62]. Clinically, clonidine can be used at between 0.05–0.2 mg depending on the tolerability and response of the medication. Severe sedation at night and morning fatigue are common side effects. However, in the case of rapid metabolizers, rebound awakening can be observed, leading to early morning awakening and inability to return to sleep. The intermittent awakening or rebound can sometimes be mitigated by a longer-acting preparation—Kapvay (Clonidine CR). Clonidine is often preferred for children with hyperactivity and inability to settle before bedtime. It does not have any known interaction with ADHD medications (both stimulants and non-stimulants).

#### 4.2.3. Iron Supplementation

Given the association of low ferritin (<75 mcg/L) with PLMD and RLS, iron supplementation may be beneficial in children with ASD and ADHD who display increased motor symptoms in sleep and low ferritin levels [42,63]. Most of the children with low ferritin are not anemic, and hence specifically obtaining ferritin levels is suggested. If the ferritin levels are low, 3 mg to 6 mg elemental iron/kg/day dose for three months could be tried [42,64,65]. It is suggested to closely monitor gastrointestinal side effects such as constipation and stomachaches, which are common with iron supplementation, especially for children with limited verbal capacity and deficits in emotional reciprocity.

## 5. Conclusions

Children and adolescents with combined ADHD and ASD have more severe symptoms across all domains. The factors responsible for intrinsic sleep problems and comorbid sleep disorders have an additive effect on the severity of sleep-related presentation in this group. The presence of untreated sleep problems can further worsen the core symptoms of individual disorders, leading to significant functional impairment and poor prognosis. It is imperative that the interventions used for individual disorders be appropriately adapted by taking into consideration the unique needs of the combined ADHD and ASD group. There is a scarcity of objective and subjective studies to better characterize the sleep problems in the newly permitted diagnostic category of combined ADHD and ASD. Future research should focus on continued exploration and definition of the characteristics of sleep problems, as well as on treatment approaches that would be most suitable in this population.
